# Avidity of pertussis toxin antibodies following vaccination with genetically versus chemically detoxified pertussis toxin-containing vaccines during pregnancy

**DOI:** 10.3389/fimmu.2025.1569151

**Published:** 2025-05-22

**Authors:** Bahaa Abu-Raya, Giuseppe Del Giudice, Anita H. J. van den Biggelaar, Yuxiao Tang, Niranjan Bhat, Hong Thai Pham, Wassana Wijagkanalan

**Affiliations:** ^1^ Canadian Center for Vaccinology, Dalhousie University, Izaak Walton Killam (IWK) Health Centre and the Nova Scotia Health Authority, Halifax, NS, Canada; ^2^ Department of Pediatrics, Dalhousie University, Halifax, NS, Canada; ^3^ Department of Microbiology and Immunology, Dalhousie University, Halifax, NS, Canada; ^4^ BioNet-Asia, Bangkok, Thailand; ^5^ Center for Vaccine Innovation and Access, Seattle, WA, United States

**Keywords:** pertussis, avidity, pertussis toxin, genetically inactivated, recombinant vaccine, maternal immunization, vaccination during pregnancy

## Abstract

**Background:**

Both the quantity and quality of circulating anti-pertussis toxin antibodies are important for protection against severe pertussis. We compared the avidity of PT-IgG antibodies in pregnant women and their infants following vaccination during pregnancy with pertussis vaccines containing genetically-detoxified pertussis toxin (PT_gen_) or chemically-detoxified PT (PT_chem_).

**Methods:**

We analyzed serum samples collected earlier from pregnant women (at delivery) and their infants (at birth and 2 months of age) participating in a clinical trial where pregnant women had been vaccinated during pregnancy with recombinant acellular pertussis vaccine containing 1 µg PT_gen_ (standalone, ap1_gen_, [n=37], or combined to tetanus and diphtheria, Tdap1_gen_ [n=34]), 2 µg PT_gen_ (Tdap2_gen_, n=35), or 5 µg PT_gen_ (TdaP5_gen_, n=34), or acellular pertussis vaccine containing 8 µg PT_chem_ (Tdap8_chem_, n=35). Avidity was assessed by adding increasing concentrations (0.25, 0.5, 1, 1.5, 2, and 3 M) of NH_4_SCN as a bond-breaking agent and measuring PT-IgG levels by ELISA.

**Findings:**

Compared with Tdap8_chem_, TdaP5_gen_ vaccination was associated with significantly higher total absolute avidity (p<0.001) and medium-high to very-high avidity PT-IgG levels (p≤0.02) in mothers at delivery, infants at birth and infants at 2 months of age. Avidity was comparable to Tdap8_chem_ after vaccination with the low-dose PT_gen_ formulations (ap1_gen_, Tdap1_gen_ or Tdap2_gen_). There were no differences for vaccination during the 2^nd^ or 3^rd^ trimester of pregnancy.

**Interpretation:**

Compared with chemically detoxified vaccines, vaccination during pregnancy with recombinant genetically detoxified acellular pertussis vaccine at lower PT concentration provides infants with at least similar or higher quality PT-IgG antibodies. Consequently, recombinant pertussis vaccines may offer comparable or better protection against pertussis.

## 1 Introduction

Pertussis is a highly contagious human respiratory infection caused by the bacterium *Bordetella pertussis.* Despite high vaccination coverage, the incidence of pertussis has been increasing globally with cyclic epidemics occurring every 2 to 5 years (1). In 2024, many countries reported the largest pertussis outbreaks since decades (2–5). Pertussis is most severe in young infants who are too young to be vaccinated (6, 7). Pertussis vaccination during pregnancy is a safe and effective strategy to protect vulnerable young infants from severe pertussis (8–11).

Pertussis toxin (PT) plays a fundamental role in the pathogenesis of pertussis (12–14) and is a component of all acellular pertussis vaccines (15–17). Especially in young infants, anti-PT antibodies are an important mechanism of protection against severe disease, which depends on both the quantity and quality of the antibody response (18–21).

Pertussis toxin must be inactivated before it can be safely administered to humans. In most acellular pertussis vaccines, PT has been chemically detoxified; however, chemical treatment can cause conformational changes that lead to dominant immunity against nonprotective epitopes (22–26). Recombinant acellular pertussis vaccines using DNA technologies introducing substitutions in the S1 subunit of wild type PT to inactivate PT were successfully developed and used in childhood immunization programs (27, 28). Genetically detoxified PT (PT_gen_) retains an antigenic conformation similar to native PT with preservation of epitopes involved in toxin-neutralization (26, 27, 29). In recent years several programs for the development of recombinant acellular pertussis booster vaccines have been initiated (30–33). Results from various clinical trials involving adolescents, adults, and pregnant women and their infants, consistently show that vaccination with PT_gen_ elicits higher PT-IgG antibody titers compared with chemically detoxified PT (PT_chem_) (30, 31, 34–37).

Avidity, which is a measure of the binding strength between an epitope and an antibody’s binding site, is an important parameter of the functionality of antibodies. Higher PT-IgG avidity may contribute to a higher capacity to neutralize pertussis toxin and protect against severe disease (38, 39). PT-IgG avidity following vaccination with chemically inactivated acellular pertussis vaccines has been studied in different populations including in infants born to mothers who were vaccinated during pregnancy (40–42), but to our knowledge has never been studied for recombinant pertussis vaccines containing PT_gen_.

In this study we compared PT-IgG avidity in pregnant women and their infants following vaccination during pregnancy (20–33 weeks gestation) with one of four different formulations of a recombinant acellular pertussis vaccine containing variable amounts of PT_gen_ compared with chemically inactivated acellular pertussis booster vaccine. A wide range of concentrations of chaotropic (bond-breaking) agent was used to allow a comprehensive analysis of PT-IgG avidity (43).

## 2 Materials and methods

### 2.1 Study design

As an exploratory objective of a phase 2 randomized controlled trial of pertussis vaccination during pregnancy, the avidity of PT-IgG antibodies was assessed in serum samples collected from a pre-selected subset of participating maternal-infant pairs. The study design, safety and immunogenicity outcomes have been reported previously (Thai Clinical Trials Registry, TCTR20180725004) (36, 37). Briefly, a total of 400 healthy pregnant women (18–40 years old) living in Bangkok, Thailand, were enrolled between February and October 2019. Participating pregnant women were randomized 1:1:1:1:1 to receive during pregnancy (at 20–33 weeks gestation) one dose of one of five study vaccines, including four recombinant acellular pertussis vaccine formulations (see: Study vaccines). Individual vaccination histories were not available, but assuming participants followed the Thai national immunization program that has had a 99% coverage for 3 childhood doses since 1996, participants likely received 3 doses of whole cell pertussis containing vaccine during childhood (44). Women who had received diphtheria, tetanus or pertussis-containing vaccine(s) within 1 year prior to enrolment were excluded.

### 2.2 Study vaccines

Recombinant pertussis vaccines were produced by BioNet-Asia (Thailand). PT_gen_ was produced from a recombinant *B. pertussis* strain containing a substitution of two amino acids (R9K and E129G) at the enzymatic active site in sub-unit S1 in the PT operon (29). Formulations included: ap1_gen_ containing 1 µg PT_gen_ and 1 µg filamentous hemagglutinin (FHA); Tdap1_gen_ containing tetanus toxoid (7.5 Lf) and reduced-dose diphtheria toxoid (2 Lf) (Td) combined with ap1_gen_; Tdap2_gen_ (Boostagen_RED_^®^) containing 2 µg PT_gen_ and 5 µg FHA combined with Td; a licensed TdaP5_gen_ (Boostagen^®^) 5 µg PT_gen_ and 5 µg FHA combined with Td. The licensed Tdap8_chem_ comparator (Boostrix™, GlaxoSmithKline) contained 8 µg PT_chem_, 8 µg FHA and 2.5 μg pertactin combined with 5 Lf tetanus toxoid and 2.5 Lf diphtheria toxoid.

### 2.3 Ethical consideration

The clinical study was conducted in compliance with the International Council for Harmonisation of Technical Requirements for Pharmaceuticals for Human Use (ICH) and Good Clinical Practice (GCP), the Declaration of Helsinki, and local ethical guidelines. Ethical approval was obtained from the Institutional Review Boards of the Faculty of Medicine Siriraj Hospital at Mahidol University, Faculty of Medicine at Chulalongkorn University, Bangkok, Thailand and Western Institutional Review Board (now known as WIRB-Copernicus Group), Washington, USA. Written informed consent was obtained from all pregnant women before recruitment, including consent for follow-up of their newborns.

### 2.4 Samples collection and processing

Venous blood samples were randomly selected as a subpopulation of cohort samples obtained from pregnant women at the time of delivery, and from their infants at the time of birth (cord blood or from newborn within 72 hours after birth) and at 2 months of age. Avidity was assessed for a randomly selected subset of mother-infant pairs: ap1_gen_, n=37, Tdap1_gen_, n=34, Tdap2_gen_, n=35, TdaP5_gen_, n=34 (n=33 for infants at 2 months), and Tdap8_chem_, n=35 (n=34 for infants at 2 months). Sera were stored at ≤ -20 °C before being shipped on dry ice to the University of British Columbia (Vancouver, British Columbia, Canada) for avidity testing.

### 2.5 Measurement of total PT-IgG, PT-IgG avidity, avidity indices and calculations

Avidity of PT-IgG antibodies was assessed by measuring PT-specific IgG antibody binding in the presence of a range of chaotrope concentrations (NH_4_SCN at 0.25 molar (M), 0.5M, 1M, 1.5M, 2M, 3M]) using a commercial ELISA kit (EURIMMUN) coated with native highly purified *Bordetella pertussis* toxin, as published previously (41, 43). Total PT-IgG was measured in PBS (0M NH_4_SCN) in the same ELISA kit at the same time for avidity assay. PT-IgG levels were calculated against the calibration serums quantified based on the WHO International Standard Pertussis Antiserum, human (1^st^ IS NIBSC code 06/140) according to the instruction. Different avidity indices for PT-IgG were calculated as published previously (41, 43) and as described in 2.5.1, 2.5.2 and 2.5.3, and summarized in **Table 1**.

**Table 1:** Calculation of relative avidity index, fractional relative avidity index, total relative avidity index and quantification of fractional and absolute avidity levels of anti-PT IgG.

Avidity indices	NH_4_SCN concentration							
	3 M	2 M	1.5 M	1 M	0.5 M	0.25 M	0 M	<0.25M **
PT-IgG levels (IU/mL)	T_3M_	T_2M_	T_1.5M_	T_1M_	T_0.5M_	T_0.25M_	T_0M_	N/A
Relative avidity index (RAI)^*^ (%)	RAI_3M_ = T_3M_/T_0M_*100	RAI_2M_ = T_2M_/T_0M_*100	RAI_1.5M_ = T_1.5M_/T_0M_ *100	RAI_1_ = T_1M_/T_0M_ *100	RAI_0.5_ = T_0.5M_/T_0M_ *100	RAI_0.25M_ = T_0.25M_/T_0M_ *100	N/A	N/A
Fractional RAI (%)	F RAI_3M_ = RAI_3M_	F RAI_2M_ = RAI_2M_-RAI_3M_	F RAI_1.5M_ = RAI_1.5M_-RAI_2M_	F RAI_1M_ = RAI_1M_-RAI_1.5M_	F RAI_0.5M_ = RAI_0.5M_-RAI_1M_	F RAI_0.25M_ = RAI_0.25M_-RAI_0.5M_	N/A	F RAI_<0.25M_ = 100% - RAI_0.25M_
Total RAI (AU)	(F RAI_3M_*3) + (F RAI_2M_*2) + (F RAI_1.5M_*1.5) + (F RAI_1M_*1) + (F RAI_0.5M_*0.5) + (F RAI_0.25M_*0.25) + (F RAI_<0.25M_*0.125)							
Fractional absolute avidity levels (IU/mL)	F abs_3M_ = F RAI_3M_*T_0M_	F abs_2M_ = F RAI_2M_*T_0M_	F abs_1.5M_ = F RAI_1.5M_*T_0M_	F abs_1M_ = F RAI_1M_*T_0M_	F abs_0.5M_ = F RAI_0.5M_*T_0M_	F abs_0.25M_ = FRAI_0.25M_*T_0M_	N/A	F abs <_0.25M_ = FRAI_<0.25M_*T_0M_
Total absolute avidity levels (AAU/mL)	(F abs_3M_*3) + (F abs_2M_*2) + (F abs_1.5M_*1.5) + (F abs_1M_*1) + (F abs_0.5M_*0.5) + (F abs_0.25M_*0.25) + (F abs_<0.25M_*0.125)							

PT, pertussis toxin; IgG, immunoglobulin G; M, molar; N/A, not applicable; IU/mL, international unit/ml; T, total; RAI, relative avidity index; F, fractional; AU, Avidity Unit; AAU/mL, Absolute Avidity Unit/mL; abs, absolute.

*Samples treated with 0.25M, 0.5M, 1M, 1.5M, 2M, 3M concentrations of NH_4_SCN and with optic density values lower than LLOQ in ELISA were assigned an arbitrary RAI value of 15%, 12.5%, 10%, 7.5%, 5%, 2.5% for each NH_4_SCN concentrations, respectively. The fractional absolute levels of antibodies quantified at 0.25 M, 0.5 M, 1M, 1.5 M, 2M, and 3M of chaotrope were classified as low, low-medium, medium, medium–high, high and very-high avidity antibodies, respectively. The levels of antibodies eluted by the lowest chaotrope concentration (0.25 M) were classified as very-low avidity antibodies.

**This column includes the Fractional (F) RAI and Fractional (F) absolute (abs) avidity levels of PT-IgG antibodies eluted at the lowest NH_4_SCN concentration (Reproduced with minimal changes from Abu-Raya et al, Front. Immunol. 2019).

#### 2.5.1 Total relative avidity index

The total relative avidity index (total RAI) of PT-IgG antibodies was calculated for each sample. First, a relative avidity index (RAI) was calculated for each NH_4_SCN concentration as the proportion (%) of PT-IgG concentration in samples treated versus not treated with NH_4_SCN (for example, RAI_3M_=T_3M_/T_0M_*100 where T_3M_ is PT-IgG concentrations in the presence of 3M NH_4_SCN and T_0M_ is PT-IgG concentrations in the absence of NH_4_SCN). Next, a fractional RAI (F RAI) (%), defined as the RAI achieved at a specific NH_4_SCN concentration, was calculated as the RAI at a specific concentration minus the RAI achieved at the next higher concentration of NH_4_SCN (for example, RAI_1M_=RAI_1M_-RAI_1.5M_=70%-30%=40%, where 1M and 1.5M represent increasing NH_4_SCN concentrations). Finally, for each sample the total RAI (AU), reflecting the weighted contribution of the fractional RAIs achieved at different NH_4_SCN concentrations, was calculated by applying a factor to each fractional RAI corresponding to the respective concentration of NH_4_SCN giving higher weight to antibodies with higher avidity (e.g. fractional RAI at 2M given a weight of 2): (F RAI_3M_*3) + (F RAI_2M_*2) + (F RAI_1.5M_*1.5) + (F RAI_1M_*1) + (F RAI_0.5M_*0.5) + (F RAI_0.25M_*0.25) + (F RAI_<0.25M_*0.125) as published previously (41, 43).

#### 2.5.2 Fractional absolute avidity levels

As indices involving RAI are relative measures, fractional and total absolute avidity levels were calculated. The fractional absolute avidity level (F abs) of PT-IgG (IU/mL) reflects the level of PT-IgG that is still bound to the antigen at a specific NH_4_SCN concentration and calculated as the fractional RAI at a specific NH_4_SCN concentration multiplied by the anti-PT IgG concentration in the absence of NH_4_SCN (for example, F abs_3M_ = F RAI_3M_*T_0M_). F abs quantified (bound to PT) at 0.25M, 0.5M, 1M, 1.5M, 2M, and 3M of NH_4_SCN were classified as low, low-medium, medium, medium-high, high and very-high avidity antibodies, respectively. The levels of antibodies eluted (i.e. not bound to the plate) at the lowest NH_4_SCN concentration (0.25M) were classified as ‘very-low’ avidity antibodies.

#### 2.5.3 Total absolute avidity levels

Total absolute avidity levels (AAU/mL) reflect the weighted contribution of the F abs, and higher weight was given to antibodies with higher avidity by applying a factor to each fractional absolute avidity levels corresponding to the respective concentration of NH_4_SCN: (F abs_3M_*3) + (F abs_2M_*2) + (F abs_1.5M_*1.5) + (F abs_1M_*1)+ (F abs_0.5M_*0.5) + (F abs_0.25M_*0.25) + (F abs_<0.25M_*0.125) as published previously (41, 43).

### 2.6 Statistical analysis

Statistical analyses were performed by the Center of Excellence for Biomedical and Public Health Informatics (BIOPHICS), Bangkok, Thailand, using Statistical Analysis System (SAS) version 9.4. Data was analyzed *per protocol.* As this was an exploratory analysis of the main clinical study, no formal hypothesis was generated for this study.

Samples treated with 0.25M, 0.5M, 1M, 1.5M, 2M, 3M concentrations of NH_4_SCN and with optic density values lower than LLOQ were assigned an arbitrary RAI value of 15%, 12.5%, 10%, 7.5%, 5%, 2.5% for each NH_4_SCN concentrations, respectively. Total PT-IgG levels, total absolute avidity levels of PT-IgG, and F abs levels of PT-IgG did not follow a normal distribution and were log-transformed to calculate geometric mean concentrations (GMCs) and 95% confidence intervals (95% CI). Total RAI of PT-IgG followed a normal distribution and means with 95% CI were calculated. Outcomes were compared for statistical differences between the five different vaccine groups using the Kruskal-Wallis test. In addition, differences between an individual recombinant vaccine group and Tdap8_chem_ were compared using an Independent t-test. Correlations between total PT-IgG and total RAI were assessed by calculating the Spearman correlation coefficient rho. A p-value of ≤ 0.05 was considered statistically significant.

## 3 Results

### 3.1 Study population

Demographics and baseline characteristics of pregnant women and their infants included in the avidity analysis are presented in **Supplementary Table S1**. Vaccination during the 2^nd^ (13–26 weeks gestation) vs. 3^rd^ trimester of pregnancy (≥ 27 weeks gestation) was evenly distributed amongst the vaccine groups.

### 3.2 Correlation between avidity and PT-IgG levels

Overall correlations between PT-IgG levels and total RAI across (for all vaccine groups combined) were moderate in pregnant women at delivery (Spearman rho = 0.620, p<0.0001), in infants at birth (rho = 0.526, p < 0.0001) and at 2 months of age, rho = 0.724, p<0.0001) (**Supplementary Figure S1**). This indicates that the avidity of anti-PT IgG measures a function that is not entirely dependent on anti-PT IgG levels.

### 3.3 Total relative avidity index

**Figure 1:**
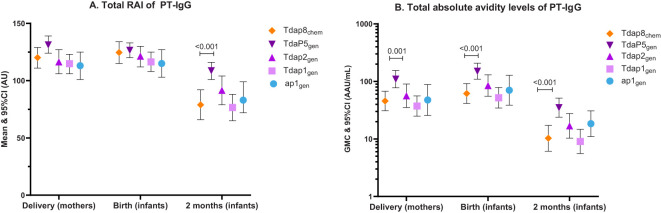
PT-IgG total relative avidity index and total absolute avidity in women at delivery, newborns at birth and infants at 2 months of age. The figure shows **(A)** means and 95% confidence intervals (CI) of PT-IgG total relative avidity (total RAI), and **(B)** geometric mean concentrations (GMC) and 95% CI of PT-IgG total absolute avidity levels in pregnant women at delivery and their infants at birth and 2 months of age after vaccination during pregnancy with Tdap8_chem_ (orange; diamond);TdaP5_gen_ (dark purple; downward triangle); Tdap2_gen_ (purple; upward triangle); Tdap1_gen_ (pink; square); or ap1_gen_ (blue; circle). For each individual recombinant vaccine group responses were compared with responses for Tdap8_chem_ using an Independent t-test: when significant (p-value ≤ 0.05), the p-value is noted.

PT-IgG total RAIs were comparable in pregnant women at delivery and in infants at birth for each of the recombinant pertussis vaccine formulations compared with Tdap8_chem_ (**Figure 1A**) (**Table 2**). However, at 2 months of age, PT-IgG total RAI was significantly higher in infants whose mothers had received TdaP5_gen_ as compared with Tdap8_chem_ (p < 0.001) (**Figure 1A**; **Table 2**).

**Table 2:** Levels of total PT-IgG, and PT-IgG total relative avidity index, total absolute avidity, and fractional absolute avidity levels of very low to very high avidities in pregnant women at the time of delivery, and infants at the time of birth and at 2 months of age after vaccination during pregnancy with different formulations of recombinant pertussis vaccines or chemically detoxified pertussis vaccine.

Pregnant women at delivery						
	ap1_gen_ (n=37)	Tdap1_gen_ (n=34)	Tdap2_gen_ (n=35)	TdaP5_gen_ (n=34)	Tdap8_chem_ (n=35)	P-value
*PT-IgG, IU/mL* GMC (95% CI)	46.88 (29.44-74.66)	33.85 (24.65-46.48)	50.96 (35.05-74.07)	85.06 (61.70-117.25)	39.58 (29.47-53.17)	0.0018
*Total RAI*, AU Mean (SD)	113.09 (36.48)	114.82 (25.08)	116.36 (31.45)	131.25 (21.96)	120.20 (27.40)	0.0468
*Total absolute avidity, AAU/mL* GMC (95% CI)	47.62 (25.65-88.42)	37.38 (24.96-55.99)	56.31 (35.18-90.15)	109.72 (77.49-155.34)	45.64 (30.90-67.42)	0.0009
*F absolute avidity, IU/mL* GMC (95% CI)						
Very low (<0.25 M) Low (0.25 M) Low-medium (0.5 M) Medium (1.0 M) Medium-high (1.5 M) High (2.0 M) Very high (3.0 M)	4.21 (2.47-7.16) 2.59 (1.44-4.67) 5.69 (3.12-10.36) 5.68 (3.03-10.64) 7.29 (3.91-13.59) 6.91 (3.24-14.74) 2.69 (1.37-5.30)	3.58 (2.48-5.17) 1.75 (1.03-2.98) 4.90 (3.31-7.27) 5.37 (3.62-7.97) 5.06 (3.37-7.58) 5.34 (2.92-9.76) 1.26 (0.75-2.11)	4.62 (3.11-6.86) 3.57 (2.55-5.00) 8.18 (5.07-13.18) 6.18 (3.69-10.35) 5.97 (3.45-10.35) 9.19 (5.06-16.67) 2.81 (1.59-4.96)	7.19 (5.25-9.85) 4.08 (2.53-6.58) 12.67 (8.68-18.51) 12.66 (8.68-18.46) 11.20 (7.42-16.91) 19.60 (13.42-28.63) 7.43 (4.68-11.77)	3.68 (2.59-5.22) 2.46 (1.66-3.64) 6.74 (4.70-9.66) 5.35 (3.65-7.84) 5.96 (4.21-8.44) 6.40 (3.66-11.19) 2.21(1.27-3.85)	0.0446 0.0818 0.0314 0.0019 0.0087 0.0020 0.0011
Infants at the time of birth						
	ap1_gen_ (n=37)	Tdap1_gen_ (n=34)	Tdap2_gen_ (n=35)	TdaP5_gen_ (n=34)	Tdap8_chem_ (n=35)	P-value
*PT-IgG, IU/mL* GMC (95% CI)	67.39 (42.77-106.19)	46.33 (33.72-63.65)	72.10 (50.45-103.06)	120.17 (88.85-162.52)	51.58 (38.30-69.47)	0.0010
*Total RAI, AU* Mean (SD)	115.00 (37.91)	116.40 (24.16)	121.40 (27.21)	126.53 (18.46)	124.65 (29.02)	0.4630
*Total absolute avidity, AAU/mL* GMC (95% CI)	70.23 (38.67-127.57)	51.92 (34.46-78.23)	84.61 (55.10-129.94)	150.31 (109.38-206.55)	61.58 (41.42-91.55)	0.0014
*F absolute avidity, IU/mL* GMC (95% CI)						
Very low (<0.25 M) Low (0.25 M) Low-medium (0.5 M) Medium (1.0 M) Medium-high (1.5 M) High (2.0 M) Very high (3.0 M)	4.85 (2.74-8.58) 3.84 (2.09-7.06) 9.61 (5.37-17.19) 7.67 (4.31-13.63) 6.88 (3.68-12.86) 12.28 (6.00-25.14) 4.51 (2.37-8.60)	5.33 (3.76-7.57) 2.43 (1.56-3.79) 6.87 (4.30-10.96) 6.93 (4.73-10.17) 5.78 (3.61-9.24) 9.05 (5.35-15.30) 2.17 (1.29-3.66)	7.43 (5.56-9.92) 4.68 (3.12-7.03) 10.26 (7.02-15.01) 9.00 (5.59-14.49) 11.22 (6.89-18.28) 13.22 (7.81-22.36) 4.50 (2.54-7.97)	11.18 (7.69-16.27) 7.33 (4.89-10.99) 18.72 (13.82-25.34) 16.47 (11.96-22.68) 18.29 (12.67-26.42) 27.57 (18.98-40.05) 9.32 (6.17-14.06)	4.39 (3.04-6.33) 3.73 (2.62-5.32) 7.31 (4.98-10.73) 6.98 (4.78-10.18) 7.28 (4.91-10.80) 9.67 (5.87-15.95) 3.69 (2.14-6.34)	0.0092 0.0332 0.0015 0.0167 0.0009 0.0006 0.0037
Infants at 2 months of age						
	ap1_gen_ (n=37)	Tdap1_gen_ (n=34)	Tdap2_gen_ (n=35)	TdaP5_gen_ (n=33)	Tdap8_chem_ (n=34)	P-value
*PT-IgG, IU/mL* GMC (95% CI)	24.96 (17.55-35.49)	13.63 (10.01-18.55)	20.95 (15.08-29.10)	33.34 (24.30-45.74)	15.30 (11.09-21.10)	0.0012
*Total RAI, AU* Mean (SD)	82.98 (33.43)	76.57 (34.79)	91.53 (37.57)	108.73 (21.46)	78.88 (38.81)	0.0011
*Total absolute avidity, AAU/mL* GMC (95% CI)	18.42 (11.02-30.79)	9.05 (5.56-14.74)	16.87 (10.29-27.67)	35.04 (23.97-51.23)	10.29 (6.13-17.26)	0.0010
*F absolute avidity, IU/mL* GMC (95% CI)						
Very low (<0.25 M) Low (0.25 M) Low-medium (0.5 M) Medium (1.0 M) Medium-high (1.5 M) High (2.0 M) Very high (3.0 M)	3.35 (2.08-5.40) 1.87 (1.07-3.25) 3.86 (2.29-6.50) 2.57 (1.41-4.68) 1.85 (1.01-3.38) 1.90 (0.96-3.78) 0.73 (0.47-1.14)	1.84 (1.09-3.11) 0.68 (0.40-1.16) 2.03 (1.15-3.59) 1.23 (0.67-2.25) 1.03 (0.56-1.90) 0.67 (0.36-1.23) 0.37 (0.25-0.55)	2.72 (1.73-4.29) 0.96 (0.59-1.57) 2.87 (1.71-4.84) 2.07 (1.18-3.65) 2.17 (1.22-3.88) 1.76 (0.91-3.42) 0.64 (0.40-1.03)	3.84 (2.77-5.32) 1.97 (1.25-3.10) 5.19 (3.60-7.48) 4.87 (3.30-7.18) 5.73 (4.08-8.06) 4.78 (2.69-8.48) 1.11 (0.68-1.81)	1.89 (1.13-3.18) 1.09 (0.66-1.81) 1.91 (1.06-3.47) 1.22 (0.67-2.25) 1.04 (0.56-1.95) 0.95 (0.49-1.83) 0.43 (0.29-0.64)	0.0547 0.0124 0.0550 0.0125 0.0003 0.0005 0.0024

RAI, relative avidity index; F, Fractional; SD, standard deviation; AU, Avidity Unit; AAU/mL, Absolute Avidity Unit/mL; ap1_gen_, acellular-pertussis vaccine containing 1 µg of pertussis toxin genetically detoxified (PT_gen_); Tdap1_gen_, tetanus, reduced-dose diphtheria [Td] combined with ap1_gen_; Tdap2_gen_, Td combined with 2 µg PT_gen_; TdaP5_gen_, Td combined with 5 µg PT_gen_; Tdap8_chem_ Td combined with 8 µg of pertussis toxin chemically-detoxified; PT, Pertussis toxin; IgG, immunoglobulin G; IU, international unit; GMC, Geometric mean concentration; CI, Confidence interval. P-values are based on comparison of outcomes for all vaccine groups using Kruskal-Wallis Test. A p-value of ≤ 0.05 is considered statistically significant.

### 3.4 Total absolute avidity levels

PT-IgG total absolute avidity was significantly higher for TdaP5_gen_ compared with Tdap8_chem_ in pregnant women at the time of delivery (p = 0.0011), in infants at the time of birth (p = 0.0006), and in infants at 2 months of age (p = 0.0002) (**Figure 1B**) (**Table 2**). PT-IgG total absolute avidity after vaccination with the lower-dose recombinant pertussis vaccines (ap1_gen_, Tdap1_gen_ or Tdap2_gen_) was comparable with Tdap8_chem_ (**Figure 1B**; **Table 2**).

### 3.5 Fractional absolute avidity (very-low to very-high avidity)

**Figure 2:**
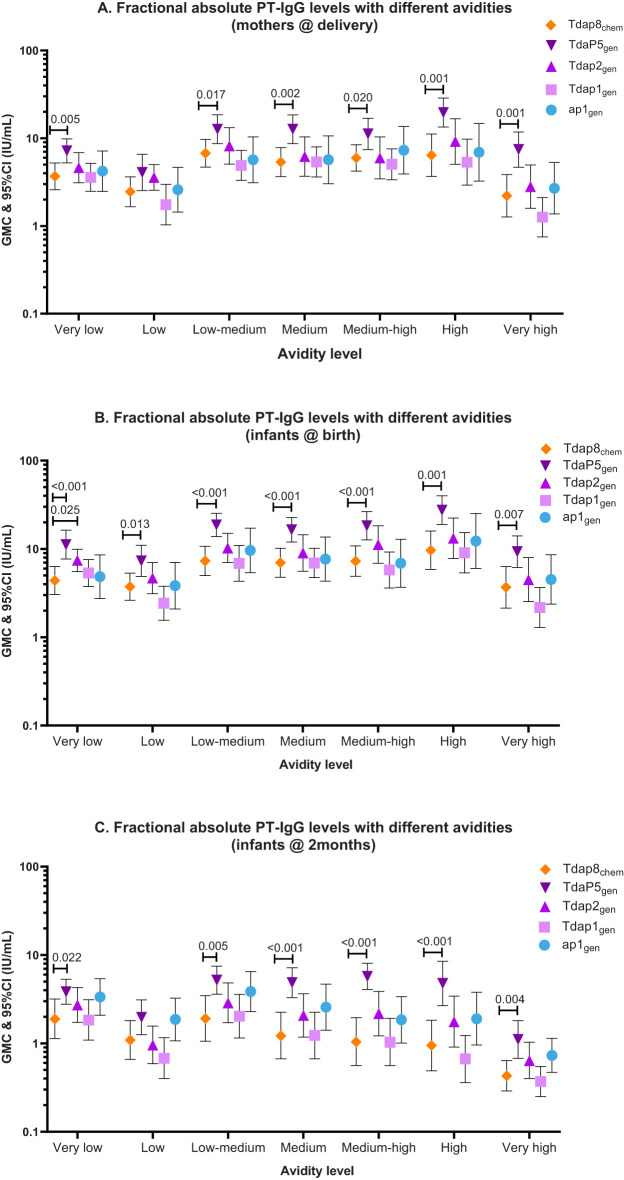
PT-IgG fractional absolute avidity (F abs) levels in women at delivery, newborns at birth and infants at 2 months of age. The figure shows geometric mean concentrations (GMC) and 95% confidence intervals (CI) for PT-IgG fractional absolute levels (F abs) with different avidities in **(A)** pregnant women at delivery, and **(B)** their infants at birth and **(C)** 2 months of age after vaccination during pregnancy with Tdap8_chem_ (orange; diamond); TdaP5_gen_ (dark purple; downward triangle); Tdap2_gen_ (purple; upward triangle); Tdap1_gen_ (pink; square)); or ap1_gen_ (blue; circle). F abs quantified at 0.25M, 0.5M, 1M, 1.5M, 2M, and 3M of NH_4_SCN were classified as low, low-medium, medium, medium-high, high and very-high avidity, respectively. Responses were compared For each individual recombinant vaccine group with responses for Tdap8_chem_ using an Independent t-test: when significant (p-value ≤ 0.05), the p-value is noted.

PT-IgG antibodies of absolute very-low to very-high avidity were comparable at the time of delivery in women vaccinated with ap1_gen_, Tdap1_gen_ or Tdap2_gen_ as compared with Tdap8_chem_, except for significantly higher levels of very-low avidity antibodies in infants at birth after vaccination in pregnancy with Tdap2_gen_ versus Tdap8_chem_ (**Figures 2A-C**; **Table 2**). Vaccination with TdaP5_gen_ was associated with significantly higher fractional absolute avidity of PT-IgG of all strengths including medium-high, high and very-high binding strength, in pregnant women at delivery, infants at birth, and infants at 2 months of age as compared with Tdap8_chem_ vaccination, with the exception of PT-IgG of low avidity which was comparable in women at delivery and infants at 2 months of age (**Figures 2A-C**; **Table 2**).

### 3.6 Effect of gestation age at the time vaccination on PT-IgG avidity

**Figure 3:**
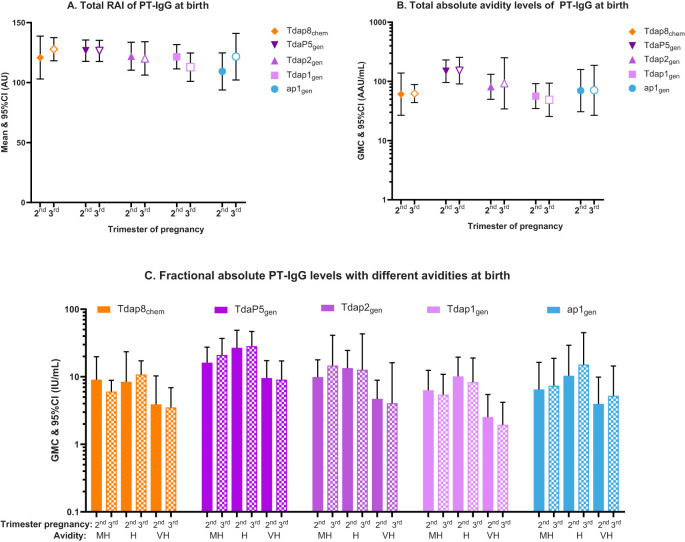
Total relative avidity index (Total RAI), total absolute avidity levels, and fractional absolute PT-IgG levels measured in newborns at the time of birth according to vaccination in the 2^nd^ or 3^rd^ trimester of pregnancy. The figure shows **(A)** means and 95% confidence intervals (CI) of PT-IgG total relative avidity (total RAI), **(B)** geometric mean concentrations (GMC) and 95% CI of PT-IgG total absolute levels, and **(C)** GMCs and 95% CI of fractional absolute levels (F abs) in infants at the time of birth after vaccination with Tdap8_chem_ (orange; diamond); TdaP5_gen_ (dark purple; downward triangle); Tdap2_gen_ (purple; upward triangle); Tdap1_gen_ (pink; square); or ap1_gen_ (blue; circle) during the 2^nd^ trimester (closed symbols and bars) or 3^rd^ trimester (open symbols and pattern bars). Only F abs quantified at 1.5M, 2M, and 3M of NH_4_SCN and classified as medium-high (MH), high (H) and very-high (VH) avidity, are presented. Responses were compared for vaccination during the 2^nd^ versus. 3^rd^ trimester using an Independent t-test, but no statistical differences were found.

No differences in PT-IgG avidity were observed in any of the vaccine groups when comparing vaccination in the 2^nd^ versus 3^rd^ trimester of pregnancy. This includes total RAI, total absolute avidity levels, and fractional absolute PT-IgG levels at delivery, in infants at birth and 2 months of age (**Figures 3A-C**; **Supplementary Table S2**).

## 4 Discussion

To our best knowledge, this is the first study describing avidity for PT-IgG antibodies induced by recombinant acellular pertussis vaccine containing genetically inactivated PT. TdaP5_gen_, a licensed recombinant acellular pertussis vaccine containing 5 µg genetically inactivated PT, induced significantly higher PT-IgG avidity in pregnant women and transferred to their infants than a widely used Tdap booster containing 8 µg chemically detoxified PT. Vaccination with lower-dose recombinant acellular pertussis vaccines induced comparable PT-IgG avidity in pregnant women and infants than the comparator Tdap_chem_ vaccine while containing 4- or 8- times less PT.

Several studies have reported on PT-IgG avidity after vaccination with conventional chemically detoxified acellular pertussis vaccines, including three studies analysing cord blood samples from cohorts following vaccination during pregnancy (40, 41, 45). These studies did not follow infants prospectively to investigate the persistence of PT-IgG avidity during the first months of life. In our study, we assessed PT-IgG avidity in cord but also in corresponding samples of mothers at the time of delivery, and longitudinally in infants at 2 months of age. We demonstrated the effective transplacental transfer of PT-IgG avidity from vaccinated mothers to newborns for all pertussis vaccines. Subsequent follow-up of infants at 2 months of age demonstrated that PT-IgG avidity remained significantly higher when mothers had been vaccinated with recombinant TdaP5_gen_.

A plausible explanation why genetically inactivated PT is associated with higher PT-IgG avidity is that in contrast to chemical detoxification of PT that leads to varying degrees of denaturation and loss of important protective conformational epitopes, site-specific genetic detoxification maintains the three-dimensional structure of the toxin (22, 26, 33). The crystal structure of PT_gen_ R9K/E129G (included in the recombinant pertussis vaccines studied here) is nearly identical to that of native PT and antigen stimulation of human whole blood indicated broader immunogenicity of PT_gen_ R9K/E129G compared with PT_chem_ (33). Furthermore, using cryo-electron microscopy it was recently shown that two potently neutralizing anti-PT antibodies with complementary mechanisms, hu11E6 and hu1B7, bind to PT_gen_ R9K/E129G, thereby confirming the preservation of these neutralizing binding sites in PT_gen_ (26).

Analysis of epitope binding, PT-neutralizing antibodies, memory-B cells, and avidity are all parameters of the quality of the anti-PT immune response. It has been demonstrated in multiple clinical trials that vaccination with genetically detoxified PT induces higher PT-neutralizing antibody titers compared with licensed Tdap_chem_ vaccines (31, 34, 35, 46). Longitudinal follow-up studies of participants vaccinated with recombinant acellular pertussis vaccine containing 5 µg PT_gen_ have shown that PT-neutralizing antibody levels remain elevated for at least 5 years (47, 48), and following vaccination during pregnancy PT-neutralizing antibodies are effectively transferred to infants in whom they remain elevated for at least 2 months at significantly higher levels compared with Tdap_chem_ (36, 37). Vaccination with PT_gen_ but not PT_chem_ also elicits robust memory B-cell responses to PT as demonstrated in a clinical trial of booster vaccination in adolescents (34). The current observations of higher PT-IgG avidity add further evidence to the higher quality of the immune response induced by genetically as compared with chemically detoxified PT (38, 49, 50).

This study further showed that recombinant vaccine containing lower quantities of PT_gen_ (1 µg and 2 µg) elicited PT-IgG avidity comparable to Tdap_chem_ containing 4- or 8- fold more PT. This provides further evidence that the inactivation process of PT is a critical determinant of PT-IgG avidity and that genetic versus chemical detoxification leads to higher avidity.

We also studied whether vaccination at various stages of gestation of pregnancy might affect the avidity of antibodies transferred to infants. No differences in PT-IgG avidity were observed when mothers were vaccinated during the 2^nd^ or 3^rd^ trimester of pregnancy for any of the studied vaccines. This is consistent with a Swiss study that analyzed cord blood samples from infants born to mothers vaccinated with Tdap_chem_ during pregnancy, and did not find PT-IgG avidity when comparing second versus third trimester vaccination, or different intervals between vaccination and birth (51). A difference with our study is that in the Swiss study PT-IgG avidity was assessed using three concentrations of NH_4_SCN (1M, 2M and 3M) as compared with six in our study (0.25M, 0.5M, 1M, 1.5M, 2M and 3M) and results were presented in relative avidity indices that do not incorporate absolute antibody levels. In another study using a series of chaotropic concentrations similar to our study, PT-IgG avidity was found to be higher in newborns when mothers had been vaccinated with Tdap_chem_ during 28–32 weeks of gestation as compared with 33–36 weeks of gestation, or when vaccinated 5–12 weeks before delivery versus within 4 weeks before delivery (41). It is plausible that using a broader range of chaotropic concentrations provides deeper insights into avidity development, thereby increasing the likelihood of detecting differential avidity responses. Other factors that may explain discrepancies in reported results include but are not limited to differences in pertussis epidemiology and vaccination history and the small sample size in this study.

Although there is no direct evidence confirming the clinical relevance of PT-IgG avidity, there is evidence from other respiratory bacterial infections supporting the notion that higher avidity provides higher protection. For example, in mice the levels of anti-pneumococcal serotype 6B-specific antibodies needed to prevent lethal bacteremia from the same serotype were found to be lower for high avidity antibodies (38). For *Haemophilus influenzae* type b (Hib) the avidity of antibodies induced following vaccination with Hib conjugate vaccine was shown to be a surrogate for protective immunity (49). Therefore, it may be assumed that the higher PT-IgG avidity response induced by vaccination with PT_gen_ containing vaccine contributes to improved protection compared to PT_chem_. While there are no efficacy trials for the current new generation of recombinant acellular pertussis vaccines, it has previously been reported that the efficacy of a former pediatric recombinant acellular pertussis vaccine was comparable to that of chemically detoxified acellular pertussis vaccine whilst containing 5-times less PT (50). In our study, formulations of recombinant pertussis vaccine with 4-to-8-times less PT content than the chemically detoxified comparator induced similar PT-IgG avidity, which may translate into similar efficacy.

Our study has strengths and limitations. Our study is unique in that it provides detailed characterization of a full spectrum of avidity of PT-IgG for different PT_gen_ doses and formulations of recombinant acellular pertussis vaccine. Using a dilution series of NH_4_SCN to provide the whole spectrum of avidity is essential considering the lack of knowledge of a clinically relevant levels avidity (42). In addition, antibody avidity was not only assessed in infants at birth, but also at 2 months old, and in the vaccinated mothers. This makes it one of the most comprehensive studies on PT-IgG antibody avidity following pertussis vaccination in pregnancy. Longer follow-up of infants beyond 2 months of age would have enabled demonstrating the persistence of elevated PT-IgG avidity and potential longer-lasting protection offered by maternal Tdap5_gen_ vaccination in infants; however, infants received childhood DTP vaccines starting at 2 months of age and assessing PT-IgG avidity in children following primary immunization was out of the scope of this study. In the main clinical trial, however, it was demonstrated that at 5 months of age (1 month after infants had completed the 2^nd^ priming dose), PT-IgG levels remained significantly higher in infants whose mothers had received TdaP5_gen_ versus Tdap8_chem_: a difference that may be explained by the persistence of higher maternal PT-IgG levels in the maternal recombinant TdaP5_gen_ vaccine group (52).Assessing PT-IgG avidity in infants where the local recommendation is to start the first priming dose at 3 months of age or older, may be something to consider for a future study. Other limitations include the relatively small sample size which could affect the generalizability of the results and limit statistical power, and that we did not analyze PT-IgG avidity in baseline samples in pregnant women before vaccination. Pre-vaccination PT-IgG levels had been assessed earlier and found to be low in all study groups (36): measurement of avidity would not have yielded quantifiable levels that can be analyzed. It is also yet to be studied how vaccination history may impact avidity responses. Like most pregnant women worldwide, including in countries that changed to priming with acellular pertussis vaccines, pregnant women participating in our study were vaccinated in childhood with whole cell pertussis vaccines and are unlikely to have received pertussis booster vaccines after priming in infancy (44). Studies in forthcoming years, when relatively more pregnant women will have vaccinated exclusively with acellular pertussis vaccines, may show how this affects PT-IgG antibody avidity in infants of mothers vaccinated in pregnancy.

In conclusion, the method that is used to inactivate PT for immunization influences PT-IgG avidity. Vaccination during pregnancy with recombinant acellular pertussis vaccines containing genetically detoxified PT at lower content than acellular pertussis vaccines containing chemically detoxified PT results in efficient transplacental transfer of at least similar or higher quantity and quality anti-PT antibodies. Vaccination with recombinant acellular pertussis vaccine may therefore provide infants with highly efficient and longer-lasting immune protection during the first most vulnerable months in life, but this remains to be studied.

## Data Availability

The data that support the findings of this study are available from the corresponding author upon request.
