# Supplementary material for: Avidity of pertussis toxin antibodies following vaccination with genetically versus chemically detoxified pertussis toxin-containing vaccines during pregnancy
Source: Front Immunol. 2025 May 22;16:1569151. doi: 10.3389/fimmu.2025.1569151 (PMC12137321; doi:10.3389/fimmu.2025.1569151)
Supplement: Supplementary file 1 [file DataSheet1.docx]

**Supplementary Table 1: Baseline demographic characteristics of pregnant women and their infants, and timing of vaccination in pregnancy by vaccine groups**

|  | **ap1_gen_**  **(n=37)** | **Tdap1_gen_**  **(n=34)** | **Tdap2_gen_**  **(n=35)** | **TdaP5_gen_**  **(n=34)*** | **Tdap8_chem_**  **(n=35)** |
| --- | --- | --- | --- | --- | --- |
| **Baseline demographic characteristics of pregnant women at time of recruitment** | | | | | |
| **Age, years**  Mean (SD)  *P-value comparison to Tdap_chem_ ^1^* | 30.3 (5.3)  *P=0.847* | 30.7 (4.4)  *P=0.594* | 30.6 (5.6)  *P=0.690* | 29.4 (5.7)  *P=0.593* | 30.1 (5.2)  - |
| **Height, cm**  Mean (SD)  *P-value comparison to Tdap_chem_ ^1^* | 156.9 (5.5)  *P=0.026* | 159.1 (5.6)  *P=0.482* | 159.3 (5.1)  *P=0.551* | 158.9 (5.7)  *P=0.423* | 160.1 (6.4)  - |
| **Weight, kg**  Mean (SD)  *P-value comparison to Tdap_chem_ ^1^* | 61.0 (10.2)  *P=0.121* | 66.7 (10.8)  *P=0.437* | 67.2 (12.3)  *P=0.366* | 66.3 (14.0)  *P=0.589* | 64.7 (9.9)  - |
| **Timing of vaccination in pregnancy**  2^nd^ trimester, (n [% of total])  3^rd^ trimester (n [% of total])  *P-value comparison to Tdap_chem 2_* | 20 (54.1)  17 (45.9)  *P=0.479* | 14 (41.2)  20 (58.8)  *P=0.704* | 24 (68.6)  11 (31.4)  *P=0.053* | 18 (52.9)  16 (47.1)  *P=0.548* | 16 (45.7)  19 (54.3)  - |
| **Infants’ characteristics** | | | | | |
| **Sex, male**  (n [% of total])  *P-value comparison to Tdap_chem 2_* | 17 (45.9)  *P=0.182* | 15 (44.1)  *P=0.145* | 15 (42.9)  *P=0.116* | 21 (63.6)  *P=0.874* | 21 (61.8)  - |
| **Birth Weight, gram**  Mean (SD)  *P-value comparison to Tdap_chem_ ^1^* | 5359 (629)  P=0.779 | 5164 (673)  P=0.329 | 5124 (592)  P=0.189 | 5273 (728)  P=0.789 | 5318 (613)  - |
| **Length (cm)**  Mean (SD)  *P-value comparison to Tdap_chem_ ^1^* | 57.3 (2.4)  P=0.935 | 57.1 (3.1)  P=0.751 | 57.3 (2.4)  P=0.993 | 57.4 (2.0)  P=0.954 | 57.3 (2.4)  - |
| **Head circumference, cm**  Mean (SD)  *P-value comparison to Tdap_chem_ ^1^* | 38.5 (1.4)  P=0.423 | 37.9 (1.3)  P=0.433 | 37.9 (1.6)  P=0.592 | 38.5 (1.5)  P=0.578 | 38.3 (1.3)  - |

^1^ P-values relate to testing for statistical differences of outcomes for individual PT_gen_-containing vaccines versus Tdap_chem_ using *1)* Independent t-test, or *2).* Chi-square test; *n=33 for infant data

**Supplementary Figure 1. Scatter plots of Log Total PT-IgG levels and Total relative avidity index (RAI) of PT-IgG for all vaccine group combined, in mothers at the time of delivery and in infants at time of birth and at 2 months of age**


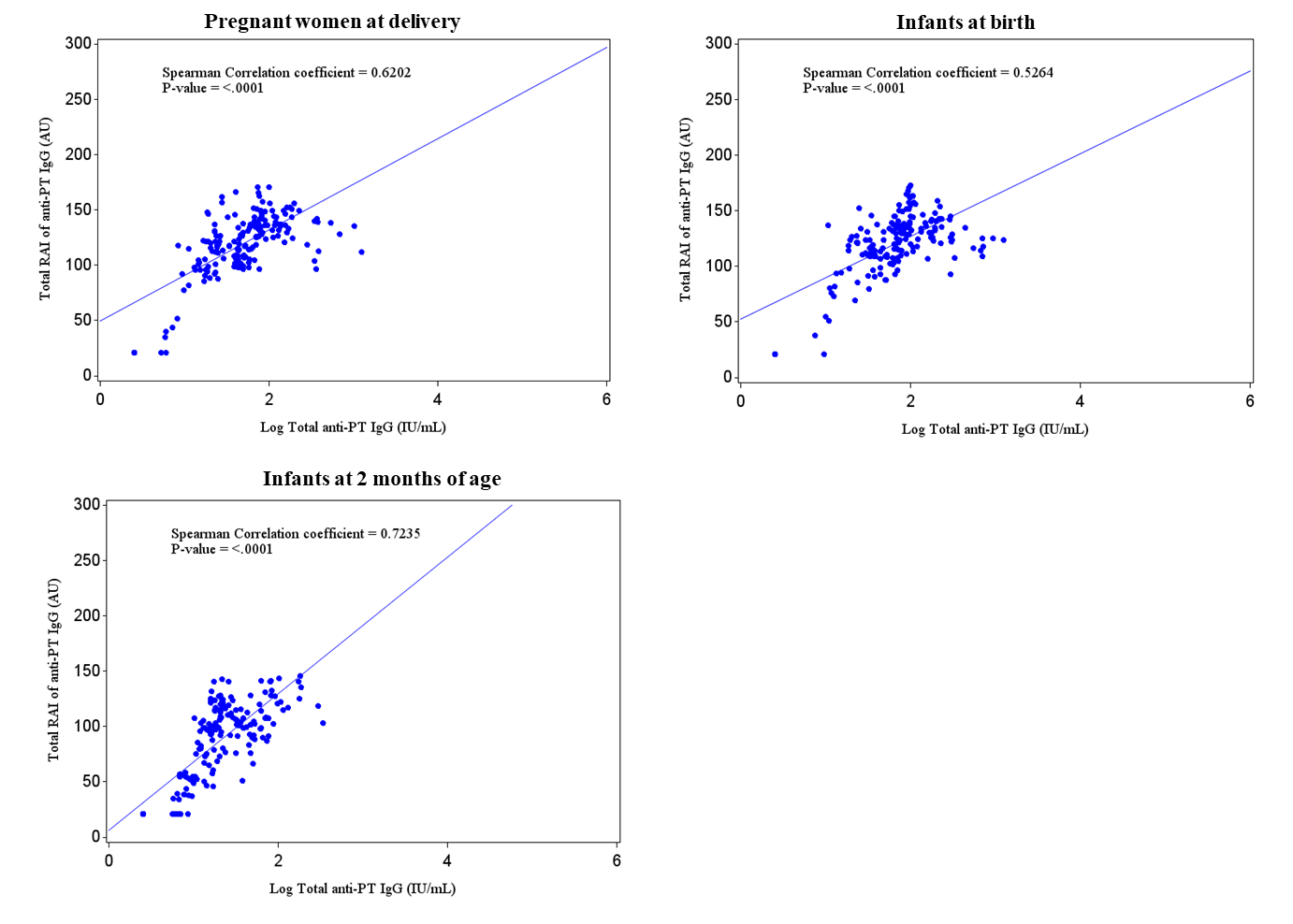


**Supplementary Table 2: Levels of total PT-IgG, and PT-IgG total relative avidity index, total absolute avidity, and fractional absolute avidity levels of very low to very high avidities in pregnant women at the time of delivery, and infants at the time of birth and at 2 months of age after vaccination during pregnancy with different formulations of recombinant pertussis vaccines or chemically detoxified pertussis vaccine administered in the 2^nd^ versus 3^rd^ trimester of pregnancy**

| 1. **Pregnant women at delivery** | | | | | | | | | | | | | | | | | | |
| --- | --- | --- | --- | --- | --- | --- | --- | --- | --- | --- | --- | --- | --- | --- | --- | --- | --- | --- |
|  | **ap1_gen_** | | | **Tdap1_gen_** | | | | | **Tdap2_gen_** | | | | **TdaP5_gen_** | | | | **Tdap8_chem_** | |
|  | Trimester of vaccination | | | Trimester of vaccination | | | | | Trimester of vaccination | | | | Trimester of vaccination | | | | Trimester of vaccination | |
|  | 2^nd^ (n=20) | 3^rd^ (n=17) | | 2^nd^ (n=14) | | 3^rd^ (n=20) | | | 2^nd^ (n=24) | | 3^rd^ (n=11) | | 2^nd^ (n=18) | | 3^rd^ (n=16) | | 2^nd^ (n=16) | 3^rd^ (n=19) |
| ***Total PT-IgG (IU/mL)*** | | | | | | | | | | | | | | | | | | |
| GMC  (95% CI) | 43.21  (22.54-82.84) | 51.60  (24.63-108.11) | | 34.03  (22.19-52.21) | | 33.72  (20.84-54.57) | | | 45.64  (32.39-64.30) | | 64.80  (22.77-184.40) | | 73.02  (47.06-113.30) | | 100.99  (60.61-168.26) | | 39.13  (22.41-68.32) | 39.97  (28.60-55.86) |
| *P-value* | *0.7065* | | | *0.9756* | | | | | *0.4941* | | | | *0.3148* | | | | *0.9455* | |
| ***Total RAI (AU)*** | | | | | | | | | | | | | | | | | | |
| Mean (SD) | 109.74  (41.34) | 117.04  (30.55) | | 117.65 (20.28) | | 112.84 (28.30) | | | 118.36  (29.42) | | 112.00  (36.62) | | 133.42  (25.91) | | 128.81  (16.97) | | 116.34 (32.33) | 123.45  (22.87) |
| *P-value* | *0.5416* | | | *0.5676* | | | | | *0.6194* | | | | *0.5397* | | | | *0.4671* | |
| ***Total absolute avidity (AAU/mL)*** | | | | | | | | | | | | | | | | | | |
| GMC  (95% CI) | 41.10  (16.44-102.73) | 56.64  (22.77-140.86) | | 39.47  (23.82-65.41) | | 35.99  (19.21-67.43) | | | 51.71  (32.61-82.02) | | 67.81  (19.03-241.70) | | 94.99  (55.57-162.38) | | 129.03  (79.77-208.69) | | 42.43  (19.43-92.67) | 48.54  (33.04-71.30) |
| *P-value* | *0.6045* | | | *0.8096* | | | | | *0.6652* | | | | *0.3742* | | | | *0.7457* | |
| ***Fractional absolute avidity (IU/mL)*** | | | | | | | | | | | | | | | | | | |
| GMC (95% CI) | | | | | | | | | | | | | | | | | | |
| Very low  (<0.25 M) | 3.70  (1.64-8.34) | 4.89  (2.33-10.28) | | 4.02  (2.81-5.74) | | 3.31  (1.82-6.03) | | | 4.24  (2.56-7.02) | | 5.57  (2.70-11.49) | | 6.18  (4.37-8.75) | | 8.52  (4.78-15.18) | | 3.55  (1.79-7.06) | 3.79  (2.63-5.46) |
| *P-value* | *0.5977* | | | *0.5617* | | | | | *0.5073* | | | | *0.3228* | | | | *0.8608* | |
| Low  (0.25 M) | 2.09  (0.92-4.76) | 3.34  (1.34-8.35) | | 1.65  (0.87-3.11) | | 1.83  (0.79-4.23) | | | 3.31  (2.30-4.76) | | 4.22  (1.85-9.64) | | 3.05  (1.59-5.84) | | 5.68  (2.70-11.97) | | 2.19  (1.03-4.64) | 2.71  (1.77-4.14) |
| *P-value* | *0.4270* | | | *0.8301* | | | | | *0.5626* | | | | *0.1901* | | | | *0.6069* | |
| Low-medium  (0.5 M) | 5.58  (2.27-13.70) | 5.82  (2.42-13.95) | | 5.90  (3.94-8.84) | | 4.30  (2.28-8.11) | | | 7.38  (4.58-11.88) | | 10.24  (2.88-36.39) | | 10.19  (6.24-16.65) | | 16.20  (8.69-30.20) | | 6.67  (3.14-14.20) | 6.80  (5.00-9.24) |
| *P-value* | *0.9448* | | | *0.3815* | | | | | *0.6026* | | | | *0.2244* | | | | *0.9617* | |
| Medium  (1.0 M) | 5.06  (2.00-12.80) | 6.46  (2.53-16.50) | | 5.80  (3.88-8.68) | | 5.09  (2.68-9.66) | | | 5.28  (3.10-9.02) | | 8.68  (2.34-32.20) | | 10.43  (5.74-18.95) | | 15.75  (9.67-25.65) | | 4.86  (2.26-10.45) | 5.80  (4.00-8.42) |
| *P-value* | *0.6987* | | | *0.7179* | | | | | *0.4527* | | | | *0.2657* | | | | *0.6616* | |
| Medium-high  (1.5 M) | 5.99  (2.24-16.00) | 9.07  (3.93-20.95) | | 4.63  (2.53-8.47) | | 5.38  (2.99-9.68) | | | 5.52  (3.25-9.37) | | 7.11  (1.58-32.06) | | 7.87  (4.41-14.03) | | 16.66  (9.27-29.95) | | 5.84  (2.81-12.11) | 6.07  (4.52-8.15) |
| *P-value* | *0.5022* | | | *0.7064* | | | | | *0.7306* | | | | *0.0624* | | | | *0.9175* | |
| High  (2.0 M) | 7.28  (2.52-21.02) | 6.51  (1.94-21.81) | | 5.80  (2.29-14.71) | | 5.04  (2.12-11.96) | | | 9.36  (5.25-16.69) | | 8.83  (1.73-45.13) | | 17.39  (8.99-33.65) | | 22.43  (15.05-33.42) | | 5.08  (1.83-14.11) | 7.79  (4.06-14.97) |
| *P-value* | *0.8829* | | | *0.8152* | | | | | *0.9417* | | | | *0.4911* | | | | *0.4599* | |
| Very high  (3.0 M) | 2.53  (1.00-6.40) | 2.89  (0.96-8.73) | | 1.32  (0.59-2.92) | | 1.23  (0.59-2.56) | | | 2.66  (1.44-4.90) | | 3.17  (0.76-13.19) | | 6.78  (3.20-14.37) | | 8.23  (4.56-14.86) | | 2.58  (0.98-6.82) | 1.93  (0.95-3.91) |
| *P-value* | *0.8441* | | | *0.8901* | | | | | *0.8061* | | | | *0.6691* | | | | *0.6113* | |
| **B. Infants at birth** | | | | | | | | | | | | | | | | | | |
|  | **ap1_gen_** | | | | **Tdap1_gen_** | | | **Tdap2_gen_** | | | | **TdaP5_gen_** | | | | **Tdap8_chem_** | | |
|  | Trimester of vaccination | | | | Trimester of vaccination | | | Trimester of vaccination | | | | Trimester of vaccination | | | | Trimester of vaccination | | |
|  | 2^nd^ (n=20) | | 3^rd^ (n=17) | | 2^nd^ (n=14) | | 3^rd^ (n=20) | 2^nd^ (n=24) | | 3^rd^ (n=11) | | 2^nd^ (n=18) | | 3^rd^ (n=16) | | 2^nd^ (n=16) | | 3^rd^ (n=19) |
| **Total PT-IgG (IU/mL)** | | | | | | | | | | | | | | | | | | |
| GMC  (95% CI) | 68.97  (36.23-131.27) | | 65.59  (32.06-134.17) | | 47.15  (31.16-71.34) | | 45.76  (28.09-74.54) | 69.27  (47.75-100.50) | | 78.69  (31.34-197.58) | | 119.17  (80.69-175.99) | | 121.30  (72.15-203.94) | | 54.40  (30.24-97.87) | | 49.33  (36.35-66.94) |
| *P-value* | *0.9130* | | | | *0.9213* | | | *0.7813* | | | | *0.9541* | | | | *0.7563* | | |
| **Total RAI (AU)** | | | | | | | | | | | | | | | | | | |
| Mean (SD) | 109.37 (35.30) | | 121.63  (40.83) | | 121.48 (19.29) | | 112.84  (26.97) | 122.00  (29.17) | | 120.11  (23.61) | | 126.61  (19.34) | | 126.43  (18.05) | | 120.86  (36.42) | | 127.84 (21.50) |
| *P-value* | *0.3402* | | | | *0.2839* | | | *0.8404* | | | | *0.9776* | | | | *0.5070* | | |
| **Total absolute avidity (AAU/mL)** | | | | | | | | | | | | | | | | | | |
| GMC  (95% CI) | 69.81  (30.75-158.49) | | 70.74  (26.79-186.75) | | 56.50  (34.86-91.58) | | 48.94  (25.63-93.44) | 81.10  (49.66-132.46) | | 92.81  (34.38-250.53) | | 148.89  (96.07-230.74) | | 151.92  (90.48-255.08) | | 60.81  (26.71-138.47) | | 62.22  (43.81-88.38) |
| *P-value* | *0.9826* | | | | *0.7090* | | | *0.7927* | | | | *0.9501* | | | | *0.9571* | | |
| **Fractional absolute avidity** (**IU/mL)** | | | | | | | | | | | | | | | | | | |
| GMC (95%CI) | | | | | | | | | | | | | | | | | | |
| Very low  (<0.25 M) | 6.33  (2.74-14.64) | | 3.54  (1.55-8.08) | | 5.42  (3.92-7.50) | | 5.27  (2.95-9.41) | 6.92  (4.92-9.74) | | 8.67  (4.64-16.18) | | 10.10  (6.24-16.32) | | 12.54  (6.60-23.85) | | 3.76  (1.79-7.88) | | 5.00  (3.56-7.02) |
| *P-value* | *0.3054* | | | | *0.9295* | | | *0.4981* | | | | *0.5699* | | | | *0.4646* | | |
| Low  (0.25 M) | 3.58  (1.51-8.48) | | 4.16  (1.60-10.84) | | 2.41  (1.33-4.40) | | 2.44  (1.24-4.79) | 4.44  (3.10-6.36) | | 5.27  (1.63-17.07) | | 6.52  (3.62-11.74) | | 8.37  (4.52-15.49) | | 4.45  (2.19-9.03) | | 3.22  (2.31-4.49) |
| *P-value* | *0.8070* | | | | *0.9833* | | | *0.7616* | | | | *0.5380* | | | | *0.3844* | | |
| Low-medium  (0.5 M) | 11.98  (5.50-26.07) | | 7.41  (2.86-19.25) | | 6.16  (3.00-12.65) | | 7.41  (3.80-14.45) | 9.33  (5.96-14.60) | | 12.63  (5.53-28.87) | | 19.95  (13.54-29.41) | | 17.42  (10.34-29.35) | | 7.08  (3.15-15.92) | | 7.50  (5.46-10.30) |
| *P-value* | *0.4171* | | | | *0.6911* | | | *0.4903* | | | | *0.6605* | | | | *0.8897* | | |
| Medium  (1.0 M) | 8.31  (3.78-18.24) | | 6.98  (2.73-17.81) | | 7.86  (5.16-11.96) | | 6.35  (3.44-11.71) | 8.58  (4.80-15.34) | | 10.00  (3.73-26.80) | | 16.56  (11.79-23.25) | | 16.37  (8.87-30.20) | | 6.17  (2.82-13.50) | | 7.74  (5.57-10.75) |
| *P-value* | *0.7652* | | | | *0.5481* | | | *0.7727* | | | | *0.9728* | | | | *0.5775* | | |
| Medium-high  (1.5 M) | 6.50  (2.58-16.35) | | 7.36  (2.89-18.74) | | 6.31  (3.20-12.45) | | 5.43  (2.72-10.84) | 9.92  (5.54-17.78) | | 14.67  (5.23-41.11) | | 16.17  (9.52-27.48) | | 21.01  (11.97-36.88) | | 9.07  (4.17-19.75) | | 6.05  (4.14-8.86) |
| *P-value* | *0.8431* | | | | *0.7448* | | | *0.4798* | | | | *0.4785* | | | | *0.3313* | | |
| High  (2.0 M) | 10.36  (3.67-29.26) | | 15.19  (5.14-44.94) | | 10.13  (5.26-19.51) | | 8.36  (3.70-18.90) | 13.47  (7.40-24.54) | | 12.69  (3.73-43.18) | | 26.88  (14.81-48.81) | | 28.37  (17.17-46.86) | | 8.45  (3.04-23.47) | | 10.83  (6.83-17.18) |
| *P-value* | *0.5935* | | | | *0.7003* | | | *0.9244* | | | | *0.8845* | | | | *0.6421* | | |
| Very high  (3 M) | 3.98  (1.61-9.88) | | 5.23  (1.90-14.42) | | 2.53  (1.17-5.45) | | 1.95  (0.91-4.18) | 4.71  (2.50-8.89) | | 4.05  (1.02-16.11) | | 9.56  (5.27-17.33) | | 9.05  (4.78-17.15) | | 3.90  (1.48-10.29) | | 3.51  (1.80-6.87) |
| *P-value* | *0.6765* | | | | *0.6160* | | | *0.8296* | | | | *0.8966* | | | | *0.8512* | | |
| **C. Infants at 2 months of age** | | | | | | | | | | | | | | | | | | |
|  | **ap1_gen_** | | | | **Tdap1_gen_** | | | **Tdap2_gen_** | | | | **TdaP5_gen_** | | | | **Tdap8_chem_** | | |
|  | Trimester of vaccination | | | | Trimester of vaccination | | | Trimester of vaccination | | | | Trimester of vaccination | | | | Trimester of vaccination | | |
|  | 2^nd^ (n=20) | | 3^rd^ (n=17) | | 2^nd^ (n=14) | | 3^rd^ (n=20) | 2^nd^ (n=24) | | 3^rd^ (n=11) | | 2^nd^ (n=18) | | 3^rd^ (n=15) | | 2^nd^ (n=16) | | 3^rd^ (n=18) |
| **Total PT-IgG (IU/mL)** | | | | | | | | | | | | | | | | | | |
| GMC  (95% CI) | 25.11  (15.35-41.07) | | 24.78  (14.13-43.48) | | 13.63  (8.70-21.35) | | 13.63  (8.65-21.48) | 19.54  (14.22-26.86) | | 24.38  (9.97-59.61) | | 32.56  (21.10-50.25) | | 34.31  (20.34-57.87) | | 17.40  (10.70-28.30) | | 13.64  (8.54-21.79) |
| *P-value* | *0.9710* | | | | *0.9996* | | | *0.6154* | | | | *0.8708* | | | | *0.4498* | | |
| **Total RAI (AU)** | | | | | | | | | | | | | | | | | | |
| Mean (SD) | 81.14 (35.42) | | 85.13  (31.85) | | 78.93  (33.34) | | 74.91  (36.53) | 92.26  (33.88) | | 89.94  (46.43) | | 109.85  (26.09) | | 107.38  (14.93) | | 86.15  (41.50) | | 72.41  (36.19) |
| *P-value* | *0.7206* | | | | *0.7422* | | | *0.8840* | | | | *0.7358* | | | | *0.3141* | | |
| **Total absolute avidity (AAU/mL)** | | | | | | | | | | | | | | | | | | |
| GMC  (95% CI) | 17.74  (8.40-37.46) | | 19.27  (8.83-42.06) | | 9.64  (4.76-19.49) | | 8.67  (4.22-17.79) | 16.33  (9.80-27.23) | | 18.10  (4.99-65.65) | | 33.85  (19.06-60.13) | | 36.53  (21.00-63.52) | | 12.76  (5.73-28.40) | | 8.49  (4.06-17.75) |
| *P-value* | *0.8728* | | | | *0.8242* | | | *0.8723* | | | | *0.8407* | | | | *0.4329* | | |
| **Fractional absolute avidity** (**IU/mL)** | | | | | | | | | | | | | | | | | | |
| GMC (95%CI) | | | | | | | | | | | | | | | | | | |
| Very low  (<0.25 M) | 3.23  (1.59-6.60) | | 3.50  (1.73-7.05) | | 2.30  (1.16-4.59) | | 1.58  (0.72-3.48) | 2.99  (1.81-4.95) | | 2.21  (0.74-6.60) | | 3.48  (2.02-5.97) | | 4.32  (2.95-6.32) | | 2.28  (1.06-4.87) | | 1.60  (0.74-3.48) |
| *P-value* | *0.8712* | | | | *0.4501* | | | *0.5904* | | | | *0.4916* | | | | *0.4993* | | |
| Low  (0.25 M) | 1.76  (0.76-4.10) | | 2.00  (0.91-4.38) | | 0.90  (0.36-2.22) | | 0.56  (0.27-1.15) | 0.95  (0.60-1.51) | | 0.98  (0.25-3.93) | | 1.72  (0.97-3.07) | | 2.30  (1.04-5.09) | | 1.37  (0.68-2.74) | | 0.90  (0.41-1.97) |
| *P-value* | *0.8220* | | | | *0.3889* | | | *0.9587* | | | | *0.5344* | | | | *0.3987* | | |
| Low-medium  (0.5 M) | 3.12  (1.38-7.05) | | 4.97  (2.52-9.83) | | 2.23  (0.97-5.14) | | 1.90  (0.82-4.37) | 3.06  (1.76-5.32) | | 2.50  (0.67-9.38) | | 4.96  (2.76-8.92) | | 5.48  (3.41-8.82) | | 1.90  (0.75-4.77) | | 1.93  (0.82-4.56) |
| *P-value* | *0.3606* | | | | *0.7702* | | | *0.7597* | | | | *0.7809* | | | | *0.9777* | | |
| Medium  (1.0 M) | 2.69  (1.17-6.15) | | 2.44  (0.93-6.42) | | 1.25  (0.47-3.29) | | 1.21  (0.52-2.84) | 1.93  (1.06-3.49) | | 2.43  (0.57-10.32) | | 4.55  (2.57-8.05) | | 5.29  (2.94-9.52) | | 1.31  (0.53-3.24) | | 1.16  (0.46-2.88) |
| *P-value* | *0.8739* | | | | *0.9600* | | | *0.7481* | | | | *0.6972* | | | | *0.8433* | | |
| Medium-high  (1.5 M) | 1.77  (0.75-4.14) | | 1.94  (0.74-5.07) | | 0.94  (0.37-2.38) | | 1.10  (0.46-2.65) | 2.13  (1.13-4.04) | | 2.25  (0.55-9.30) | | 5.20  (2.98-9.09) | | 6.45  (4.28-9.71) | | 1.32  (0.52-3.32) | | 0.85  (0.33-2.16) |
| *P-value* | *0.8770* | | | | *0.8000* | | | *0.9392* | | | | *0.5158* | | | | *0.4807* | | |
| High  (2.0 M) | 1.89  (0.71-5.00) | | 1.91  (0.65-5.66) | | 0.79  (0.29-2.16) | | 0.59  (0.26-1.36) | 1.75  (0.84-3.65) | | 1.80  (0.37-8.86) | | 5.59  (2.65-11.81) | | 3.96  (1.48-10.60) | | 1.70  (0.58-5.02) | | 0.56  (0.25-1.27) |
| *P-value* | *0.9871* | | | | *0.6467* | | | *0.9679* | | | | *0.5565* | | | | *0.0937* | | |
| Very high  (3 M) | 0.73  (0.39-1.36) | | 0.74  (0.36-1.49) | | 0.34  (0.22-0.53) | | 0.39  (0.21-0.73) | 0.56  (0.35-0.90) | | 0.85  (0.25-2.95) | | 1.12  (0.56-2.24) | | 1.09  (0.50-2.39) | | 0.52  (0.28-0.95) | | 0.37  (0.21-0.65) |
| *P-value* | *0.9824* | | | | *0.6856* | | | *0.5028* | | | | *0.9647* | | | | *0.4017* | | |

**Abbreviations:** ap1_gen_: acellular-pertussis vaccine containing 1 µg of pertussis toxin genetically detoxified (PT_gen_); Tdap1_gen_: tetanus, reduced-dose diphtheria [Td] combined with ap1_gen_; Tdap2_gen_ :Td combined with 2 µg PT_gen_; TdaP5_gen_ Td combined with 5 µg PT_gen_; Tdap_chem_ Td combined with 8 µg of pertussis toxin chemically-detoxified; PT: Pertussis toxin; IgG, immunoglobulin G; IU, international unit; GMC: Geometric mean concentration; CI: Confidence interval; RAI, relative avidity index; SD, standard deviation; AU, Avidity Unit; AAU/mL, Absolute Avidity Unit/mL.

P-values are derived from comparison of each vaccine formulation administered in 2^nd^ vs. 3^rd^ trimester and based on Independent t-test.
